# Evaluation of Preeclampsia Results after Use of Metformin in Gestation: Systematic Review and Meta-analysis

**DOI:** 10.1055/s-0038-1675214

**Published:** 2018-11-06

**Authors:** Iramar Baptistella do Nascimento, Guilherme Dienstmann, Matheus Leite Ramos de Souza, Raquel Fleig, Carla Beatriz Pimentel Cesar Hoffmann, Jean Carl Silva

**Affiliations:** 1 Department of Postgraduate in Health and Environment, Universidade da Região de Joinville, Joinville, Brazil

**Keywords:** pregnancy, metformin, treatment, preeclampsia, gravidez, metformina, tratamento, pré-eclâmpsia

## Abstract

**Objective** Does the use of metformin have an influence on the outcomes of preeclampsia (PE)?

**Sources of Data** The descriptors *pregnancy*, *metformin*, *treatment*, and *preeclampsia* associated with the Boolean operators *AND* and *OR* were found in the MEDLINE, LILACS, Embase and Cochrane databases. A flowchart with exclusion criteria and inclusion strategy using the Preferred Reporting Items for Systematic Reviews and Meta-Analyses (PRISMA) protocol, and eligibility criteria was used. Data were extracted regarding the type of study, the applied dosage, treatment time, segment, bias risks, and the Patient, Intervention, Comparison and Outcome (PICO) strategy to identify the quality of the study.

**Selection of Studies** Total number of journals in the initial search (*n* = 824); exclusions from repeated articles on different search engines (*n* = 253); exclusions after reading the titles, when the title had no correlations with the proposed theme (*n* = 164); exclusions due to incompatibility with the criteria established in the methodological analysis (*n* = 185), exclusion of articles with lower correlation with the objective of the present study (*n* = 187); and final bibliographic selection (*n* = 35).

**Data Collection** At first, a systematic review of the literature was performed. Subsequently, from the main selection, randomized and non-randomized trials with metformin that presented their results in absolute and relative numbers of PE outcomes were selected. The variables were treated statistically in the meta-analysis with the Review Manager software (RevMan), version 5.3. Copenhagen: Nordic Cochrane Centre, The Cochrane Collaboration. Denmark in the Hovedistaden region.

**Synthesis of Data** The study showed that metmorfin presented greater preventive effects for pregnancy-induced hypertension and was less effective for PE.

**Conclusion** Metformin may gain place in preventive treatments for PE, once the dosages, the gestational age, and treatment time are particularly evaluated. A methodological strategy with an improved perspective of innovative and/or carefully progressive dosages during pregnancy to avoid side effects and the possibility of maternal-fetal risks is suggested.

## Abstract (pt)

**Resumo:**

**Objetivo** O uso de metformina tem influência nos resultados da pré-eclâmpsia (PE)?

**Fontes de Dados** Os descritores *gravidez*, *metformina*, *tratamento* e *pré-eclâmpsia* associados aos operadores booleanos *AND* e *OR* foram encontrados nas bases de dados MEDLINE, LILACS, Embase e Cochrane. Foi utilizado um fluxograma com critérios de exclusão e estratégia de inclusão, utilizando o protocolo Preferred Reporting Items for Systematic Reviews and Meta-Analyses PRISMA e critérios de elegibilidade. Os dados foram extraídos quanto ao tipo de estudo, dosagem aplicada, duração do tratamento, segmento, riscos de viés e estratégia Patient, Intervention, Comparison and Outcome (PICO) para identificar a qualidade do estudo.

**Seleção de Estudos** Número total de periódicos na busca inicialmente realizada (*n* = 824); exclusões de artigos repetidos nos diferentes sites de busca (*n* = 253); exclusões após a leitura dos títulos, quando o titulo não apresentava correlações com o tema proposto (*n* = 164); exclusões por incompatibilidade com os critérios estabelecidos na análise metodológica (*n* = 185), exclusão de artigos com menor correlação com o objetivo do presente estudo (*n* = 187); e seleção bibliográfica final (*n* = 35).

**Coleta de Dados** Inicialmente, foi realizada uma revisão sistemática da literatura. Posteriormente, a partir da seleção principal, foram selecionados estudos randomizados e não randomizados com metformina, os quais apresentaram em seus resultados números absolutos e relativos de desfechos de PE. As variáveis foram tratadas estatisticamente na metanálise por meio do Review Manager software (RevMan), version 5.3. Copenhagen: Nordic Cochrane Centre, The Cochrane Collaboration. Denmark in the Hovedistaden region.

**Síntese dos Dados** O estudo demonstrou que a metmorfina apresenta maiores efeitos preventivos para a hipertensão induzida pela gravidez e é menos eficaz para a PE.

**Conclusão** A metformina pode conquistar seu espaço nos tratamentos preventivos da PE, uma vez que as dosagens, a idade gestacional e o tempo de tratamento são particularmente avaliados. Sugere-se uma estratégia metodológica com uma perspectiva aprimorada de doses inovadoras e/ou cuidadosamente progressivas durante a gravidez, a fim de evitar efeitos colaterais e a possibilidade de riscos materno-fetais.

## Introduction

Specific Hypertensive Gestation Syndromes (SHGSs) have become the object of great apprehension, both worldwide and in Brazil.^1^ According to the National High Blood Pressure Education Program Working Group on High Blood Pressure in Pregnancy (2000), these syndromes are classified as: chronic hypertension, pregnancy-induced hypertension (PIH), preeclampsia (PE), and eclampsia.^2^

Regarding PE, among the possible multifactorial causes that ratify its incidence are gestational diabetes mellitus (GDM) and weight gain above normal levels by pregnant women.^3^ ^4^ The scientific literature points to the close relationship between dyslipidemia, hyperglycemia and maternal obesity with the outcomes of PE,^3^ ^5^ since it has been one of the main causes of gestational deaths in Brazil.^1^

A recent study using metformin hydrochloride demonstrated satisfactory results in pregnancy.^3^ In addition to its pharmacokinetic action, which decreases gluconeogenesis in the liver, the drug has been successfully used in the treatment of polycystic ovary syndrome (PCOS).^6^ ^7^

In contrast, the drug presented a risk of intolerance. However, 20 randomized trials did not report serious side effects, and the impact of therapies and outcomes of the action of metmorfine during pregnancy is relatively recent.^8^

Recent studies suggest a possible decrease in specific hypertensive disease of pregnancy (SHDP), mainly a reduction in the incidence of PIH.^9^ ^10^ This fact is consistent because of the pharmacodynamics action of metformin on vascular endothelial growth factor (VEGF) related receptors and its effect of inducing a decrease in the production of angiogenic factors, improving vascular dysfunction.^3^ ^11^ ^12^

Therefore, the hypothesis was to search, through a systematic review and meta-analysis, the use of metformin in pregnancy and the consequent investigation of the statistical parameters on the outcomes of PE in the scientific literature. In this way, the present study aimed to evaluate the results of PE after the use of metformin in different treatments during pregnancy, that is, if the use of metformin has an influence on the outcomes of PE.

## Methods

A systematic review of the literature and a meta-analysis were performed between January 1, 2000, and March 30, 2018. A protocol was developed involving an evaluation report with different scientific studies. In this protocol, the 27 established items of the the Preferred Reporting Items for Systematic Reviews and Meta-Analyses (PRISMA) protocol checklist^13^ were used. Since the present study was a systematic review and a meta-analysis, the approval of an ethics committee was not required.

### Eligibility Criteria

The preference was for the researches with results of PE as primary outcome, and PIH was verified in the analysis to better understand the preventive action of metmorfine. Randomized clinical trials with the use of metformin hydrochloride in gestation were prioritized. However, other non-randomized studies with relevance to our purposes were selected if there was a higher correlation with the research objective. Among these, there were: systematic reviews and meta-analyzes; prospective cohort studies; retrospective surveys; control cases; cross-sectional studies; laboratory clinical studies; epidemiological research; studies about methods used and statistical programs.

The search for books and websites was designed by means of a selection criteria. The selected books and websites had to clarify norms, techniques and strategies to avoid bias. Consequently, a literary research was carried out in different sites of scientific research, through a flow diagram with keywords. The scientific descriptors of the Lilacs Virtual Health Library (DeCS) were used to obtain keywords. The sites used were: MEDLINE, Latin American and Caribbean Literature in the Health Sciences (LILACS), Embase and Cochrane Library databases. The descriptors *pregnancy*, *metformin*, *treatment*, and *preeclampsia*, associated with the Boolean operators *AND* and *OR* were selected, to obtain articles more adherent to the proposed theme. The final selection was composed of studies in the literature containing in their methods samples with patients who received metformin during gestation for preventive treatments of PE, or with secondary results referring to PE, or other treatments related to PCOS, GDM, and obesity.

### Selection of Studies and Extraction of Data

From the acquisition of the periodicals, three authors extracted the relevant data. It is important to note that if there was any unclear information or lack of data on the characteristics in the trials, the data, if necessary, the authors of the original articles selected could be contacted for more information.

**Fig. 1:**
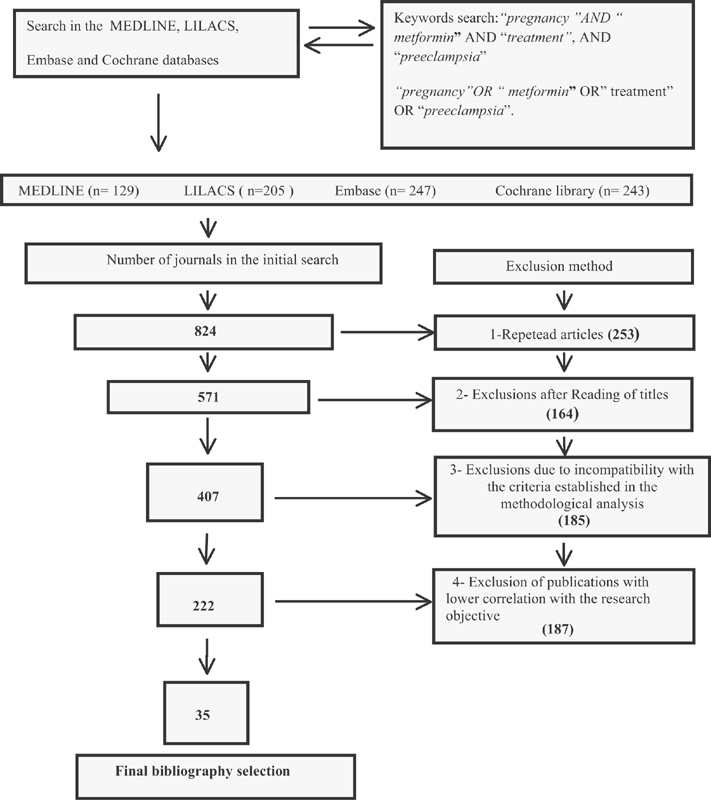
Flowchart of the activities of the selection process in the years (2000-2017).

Method of exclusion: The exclusion process was applied in relation to the following strategies: identification of repeated jobs in different search engines; reading of titles, that is, when not compatible with therapies related to the use of metformin; methodological analysis with unfilled criteria, that is, with few details in the methods section and lack of scientific record. Exclusion by objectives: when there was no consensus between purpose, method and conclusion (Fig. 1).

Inclusion criteria: article with pre-established language (Spanish, English and Portuguese). The articles should contain in the samples a population of pregnant women aged 16 years or older. We use the PICOS_14_ strategy to help in the construction of a research question and search for evidence related to the administration of metmorfin in prenatal care. Subsequently, the selection of compatible clinical trials with the meta-analysis was done. In literature review studies, the search for statistical data with metformin use in reducing PE risks was assessed. In the other studies, the samples and the methodological quality evaluation in the different studies were analyzed. The investigators independently assessed the bias risk. Three authors did the search and, if therewas doubt between two or more researchers, a fourth person of the groupwould have to do the analysis with the methodological parameters and pre-established strategies.

### Assessment of bias and methodological quality in the different studies

Attempts were made to identify the biases in selection and gauging. In this way, the following strategies were used: the research group analyzed whether a clinical trial research was in line with the pre-established objectives, that is, how the design, data collection and analysis were conducted with possible outcomes. We have tried to observe the bias of the collections by examining their data analysis and the possible impartiality in the criterion of choice, by the different scholars, in the diverse scientific researches. Consequently, the techniques used with the characteristics of the populations in the different articles were verified. Attempts were made to find the confounding effects on the outcomes obtained. The evaluations of gestational age (GA) of the research, dosages used, design and comparison of the clinical characteristics of the patients included in each study, and period of administration of metformin in the intervention groups were done in a similar way. Consequently, the search for the recent statistical values of greater impact in a comparative dynamic with other previous results was performed.

In the cohort studies, the different treatments with metformin were observed regarding the reduction in the risk of PE. Therefore, they were evaluated as follows: if the sample was adequate, if the similarity between the groups under observation existed, if they presented risks for the outcome of PE, and if the information regarding the outcomes was obtained in a similar way. Then, the similarity in both groups.

In other studies, we observed different directions that allowed complementary information and/or enriching the present research. Therefore, the same investigative properties were maintained, evaluating the particularities, reliability and validity of the data, both in the quality of the evidence in the studies selected in the systematic reviews and in the veracity of the results. As a support, the authors used the manual for systematic reviews of interventions.^15^

The main outcomes assessed were: PE outcomes in metformin versus placebo therapies in non-diabetic obese pregnant women; PE outcomes in metformin versus placebo therapies in pregnant women with PCOS; and PE outcomes in metformin versus insulin therapies in pregnant women with GDM.

The secondary outcomes assessed were: PIH outcomes in metformin versus placebo therapies in non-diabetic obese pregnant women; PIH outcomes in metformin versus placebo therapies in pregnant women with PCOS; and PIH outcomes in metformin versus insulin therapies in pregnant women with GDM.

At first, a systematic review of the literature was performed. Consecutively, from the main selection, randomized and non-randomized trials with metformin, that presented their results in absolute and relative numbers of PE outcomes were selected.

The variables were treated statistically by means of the Review Manager software (RevMan), (version 5.3. Copenhagen: Nordic Cochrane Centre, The Cochrane Collaboration. Denmark in the Hovedistaden region) in the meta-analysis. We have evaluated the primary outcomes in a fixed model using the inverse variance (IV) method. Adverse events were pooled with risk ratios (RR) using the Mantel-Haenszel method. The existence or not of heterogeneity was evaluated by the chi-squared (X^2^) test and measured by the I-squared (I^2^) test. The heterogeneity was considered significant when the X^2^ test presented *p* < 0.1 and measures of consistency, that is, high inconsistency was considered when I^2^ > 50%.^16^

When chi-squared (X^2^) *p* ≥ 0.1, it means that there was homogeneity (non-heterogenous result), that is, the heterogeneity was not significant. Therefore, *p* ≥ 0.1 means that the following differences did not occur: clinical, methodological and statistical in the studies selected for meta-analysis. When values of *p* < 0.1, it means that the heterogeneity was considered significant, that is, there were diversities between the selected studies.

Risk ratios (RR) (*P* < 0.01) The meta-analysis presented a statistically significant difference indicating a percentage of relative risk with the metformin use. When (*p* > 0.01) there was no statistically significant difference.

## Results

From the initial selection of publications added to the bases chosen and the proposed criteria, a total of 35 articles was obtained. The journals demonstrated search techniques and strategies, laboratory clinical analysis of the pharmacodynamics action of metmorfin, possible doses already applied, GA at the initiation of treatment, metformin therapies compared with placebo groups, research comparing the results of the drug with insulin during gestation, and other relevant outcomes.

Among the investigations, 8 (22.85%) were randomized clinical trials, 3 (8.57%) non-randomized clinical trials, 6 (17.14%) systematic reviews with a meta-analysis, 5 (14.28%) systematic reviews, 3 (8.57%) methodological studies and analysis techniques, 2 (5.71%) prospective cohort, 2 (5.71%) retrospective cohorts, 2 (5.71%) case controls, 2 (5.71%) epidemiological researches, and 2 (5.71%) clinical study laboratory tests (Table 1).

**Table 1:** Selected scientific studies for meta-analysis using metformin

Study and year of publication	Type of study	Dosage	Population	Segment	Country
Chiswick et al. (2015)^17^	Randomized clinical trial	500 mg/d to 2500 mg/d	Pregnant women with obese	5 to 6 months	United Kingdom
Syngelaki et al. (2016)^11^	Randomized clinical trial	500 mg/d to 3000 mg/d	Pregnant women with obese	5 to 6 months	USA and United Kingdom
Nawaz et al. (2010)^18^	Control case	1500 mg/d	Pregnant women with PCOS	5 to 6 months	Pakistan
El Hameed et al. (2011)^19^	Non-randomized clinical trial	2500 mg/d.	Pregnant women with PCOS	2 to 3 months	Egypt
Khattab et al. (2011)^10^	Prospective cohort	1000 mg/d to 1.500 mg/d	Pregnant women with PCOS	6 to 8 months	Egypt
Vanky et al. (2004)^20^	Randomized clinical trial	1700 mg/d	Pregnant women with PCOS	1 to 5 months	Norway
Vanky et al. (2010)^21^	Randomized clinical trial	2000 mg/d.	Pregnant women with PCOS	5 to 6 months	Norway
Rowan et al. (2008)^22^	Randomized clinical trial	2500 mg/d.	Pregnant women with DMG	4 to 5 months	Norway, New Zealand, and Australia
Tertti et al. (2013)^23^	Randomized clinical trial	500 mg/d to 1.000 mg/d	Pregnant women with DMG	6 to 7 months	Finland
Niromanesh et al. (2012)^24^	Randomized clinical trial	500 mg/d to 2.500 mg/d	Pregnant women with DMG	4 to 5 months	Iran

Abbreviations: DMG, gestational diabetes mellitus; PCOS, polycystic ovarian syndrome.

### Risk Ratio in Maternal Outcomes for pregnancy-induced hypertension and preemclampsia

**Fig. 2:**
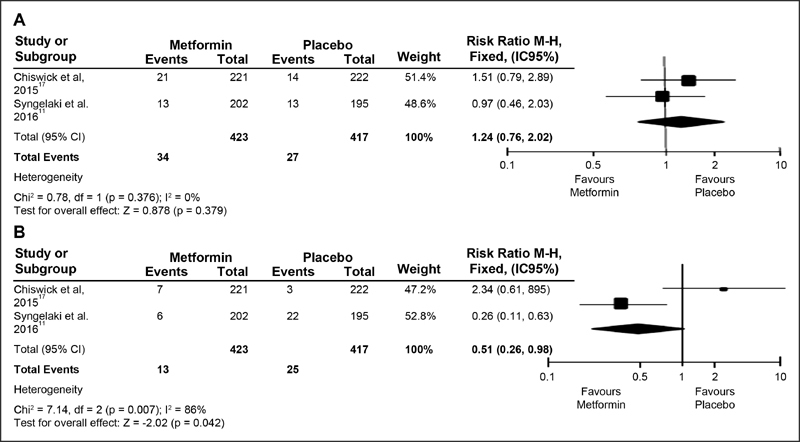
(**A** and **B**) Forest Plots. Randomized studies with obese pregnant women for HIG and PE.

The RRs grouped in the studies were as follows: randomized studies of obese pregnant women for PIH did not report significant values of risk reduction with metformin (RR = 1.24; 95% CI: 0.76–2.02; *p* = 0.379), while the researches presented homogeneity (*p* = 0.376) (*p* > 0.01) and low inconsistency (I^2^ = 0%), as shown in Fig. 2A. For the randomized studies of obese women with PE outcomes, the risk was shown to be decreased in the occurrence of disease (RR = 0.51; 95% CI: 0.26–0.98, *p* = 0.042), considering that the treatments indicated a reduction of 49% in the risk of incidence, However, they showed heterogeneity in the studies (*p* = 0.007) and high inconsistency (I^2^ = 86%), as shown in Fig. 2B.

**Fig. 3:**
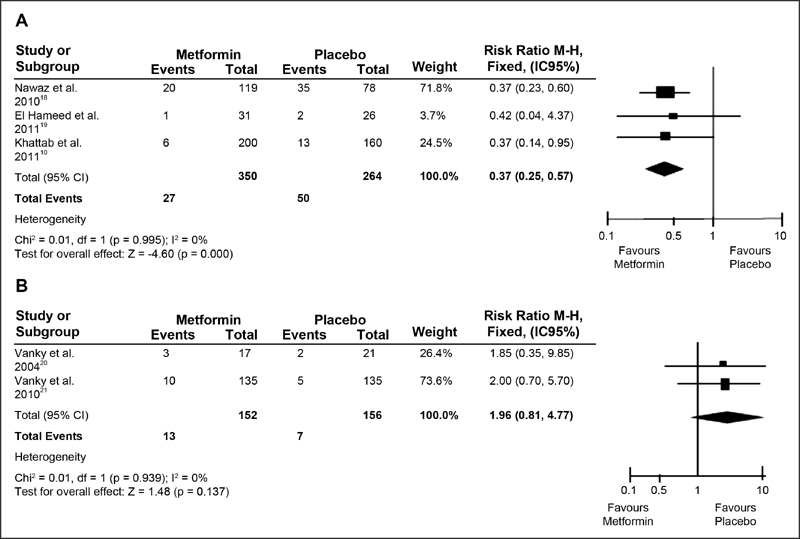
(**A** and **B**) Forest Plots. Non-randomized studies of pregnant women with PCOS for HIG and randomized studies of pregnant women with PCOS for PE.

The non-randomized investigations of pregnant women with PCOS for PIH (RR = 0.37; (95% CI: 0.25 −0.57; *p* = 0.000) (*p* < 0.01), the parameters were directed toward risk reduction for PIH with a possible 63% reduction with drug use. The researches presented homogeneity (*p* = 0.995) (*p* ≥ 0.1) and low inconsistency (I^2^ = 0%) (Fig. 3A), and for those randomized with PCOS for PE (RR = 1.96, 95% CI: 0.81 −4.77; *p* = 0.137) (*p* > 0.01), the result did not express risk reduction for PIH, indicating homogeneity in the surveys (*p* = 0.939) (*p* > 0.1) and low inconsistency (I^2^ = 0%), according to Fig. 3B.

**Fig. 4:**
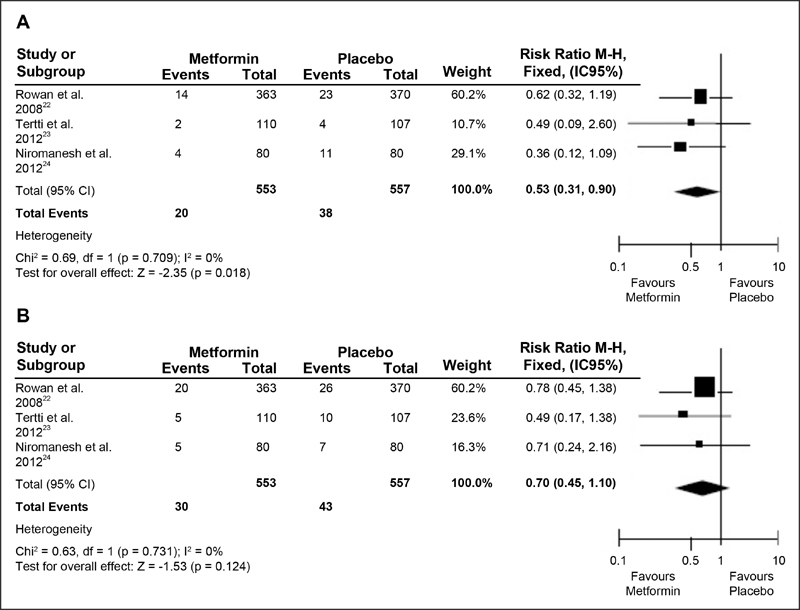
(**A** and **B**) Forest Plots. Randomized studies of pregnant women with Gestational Diabetes Mellitus (GDM) Pregnancy-induced hypertension (PIH); Preeclampsia (PE).

The randomized studies of diabetic pregnant women for PIH (RR = 0.53; 95% CI: 0.31 −0.90; *p* = 0.018) (*P* > 0.01), indicated a 47% risk reduction with metformin compared with insulin, showing homogeneity among the surveys evaluated (*p* = 0.709) (*p* > 0.1), and with low inconsistency (I^2^ = 0%); Fig. 4A. The randomized trials of diabetic pregnant women for PE (RR = 0.70; 95% CI: 0.45 −1.10; *p* = 0.124) (*P* > 0.01) showed no significant values, with homogeneity (*p* = 0.731) (*p* > 0.1) and low inconsistency (I^2^ = 0%), as shown in Fig. 4B.

For all events, the pooled studies were homogeneous (X^2;^ *p *> 0.1), except for RR of randomized studies of obese pregnant women for PE outcomes (*p* = 0.007; I^2^ = 86%), as shown in Figs. 2, 3 and 4.

## Discussion

The present study provided a brief overview of the use of metformin for the prevention of PE in research with obese patients, PCOS and GDM. The results indicated that metformin reduced PE in treatments with obese pregnant women (*p* < 0.01), but not in pregnant women with PCOS and GDM (*p* > 0.01). In PIH, the drug showed significant values for both PCOS patients and pregnant women with GDM.

Since 2009, researchers have pointed out the equivalence of metformin with insulin or its use as possible alternative treatment for GDM. However, due to its ease of passage through the placenta, it should be used with determination and caution.^23^ ^25^ Therefore, the conceivable use of metmorfin proposes a coadjutant alternative in pregnancy, since as an insulin-sensitizing agent, it has shown a reduction of GDM and maternal obesity, and, in the scientific bibliography, these intercurrences demonstrated greater associations with SHGS.^5^ ^10^ ^11^ ^26^

Regarding the prevention of PIH and PE, metformin suggests a greater attenuation of angiogenic factors, such as soluble fms-like tyrosine kinase-1 (sFlt-1) and endoglin (ENG), acting on soluble vascular endothelial growth factor receptor-1 and soluble ENG receptors, with improvements in vascular dysfunctions.^3^ ^12^ Thus, in view of the drug pharmacokinetics and pharmacodynamics action, it has been used, among others, in comparisons with placebo groups with non-diabetic pregnant women, with pregnant women with PCOS, and compared with insulin in the treatment of GDM.^17^ ^21^ ^22^

### Preeclampsia Outcomes in Metformin versus Placebo Therapies in Non-diabetic Obese Pregnant Women

In an observational cohort study, researchers identified groups with higher body mass index (BMI) (overweight and obesity) are more likely to have complications and major intercurrences related to SHGS.^5^ However, although researchers have found a reduction in weight gain of the overweight pregnant mother with metformin administration, they did not confirm significance in PIH-related outcomes.^11^

The dosage to be administered and the GA at the initiation of the treatment may be relevant in the conception of the prophylaxis of the disease. Two similar randomized trials in the current decade have shown a similarity of outcomes in PIH prophylaxis.

In the first study in 2015, the pregnant women started the administration of metformin between 12 and 16 weeks of gestation, used a dosage of 500 mg/d for 5 weeks for adaptation and, from the sixth week on, the dosage was changed to 2500 mg/d until the delivery. Since the results did not indicate significant differences for both PIH (odds ratio [OR] = 1.56; 95% CI: 0.77–3.15) and PE (OR = 2.39; CI 95%: 0.61–9.36).^17^

In the following year, another study started the intervention at between 12 and 18 weeks of gestation with 500 mg/d per week, up to a maximum dosage of 3000 mg/d in the 5^th^ week before the delivery. The study demonstrated for PIH (OR = 1.11; 95% CI: 0.60–2.04; *p *> 0.01) and, for prevention of PE, showed a difference of 3% in the metformin group compared with 11% in the placebo group (OR = 0.24; 95% CI: 0.10–0.61; *p* < 0.01).^11^ Our results in the meta-analysis indicated a reduction in the RR of PE (RR = 0.51; 95% CI: 0.26–0.98) in obese pregnant women who used metformin.

However, it is worth highlighting the need for more detailed observation on the dosages, since during pregnancy renal clearance becomes greater and metformin may be subsequently eliminated during the gestacional period.^27^

### Outcomes of Preeclampsia in Metformin versus Placebo Therapies in Pregnant Women with Polycystic Ovary Syndrome

Polycystic ovary syndrome is an endocrine disorder of anovulatory infertility in women of reproductive age. Its prevalence depends on the criteria used, taking into account the population studied.^28^ Metformin reduces androgenic hormones and also tends to correct the insulin resistance present in practically all women with PCOS.^29^ Among other complications, DMG, PIH and PE are prevalent complications in pregnant women with PCOS.^30^

A clinical trial with pregnant women with PCOS using 850 mg/d of metmorfin in the first week and 1700 mg/d for the remainder of the study period, between 5 and 12 weeks of pregnancy, showed no relevance for PE.^20^ A subsequent randomized study with similarity at the starting point for treatment, between 5 and 12 weeks of gestation, ratified non-significant values for PE (*p* < 0.01), even at a dosage of 2000 mg/d.^21^

In contrast, non-randomized studies showed a greater significance for PIH. Researches that used a treatment with 1500 mg/d of metmorfin presented a difference between the intervention groups (16.5% versus 45% [control]; *p* = 0.002).^18^ It is worth mentioning a prospective cohort in which women were taking metformin 3 to 6 months before pregnancy and the intervention group continued to administer the drug in one dosage of 1000–2000 mg/d during gestation. The results were significant: 3% of metformin group versus 6% of control group demarcating a reduction of PIH, (OR = 0.35; 95% CI: 0.13 −0.94; *p* < 0.01).^10^ In contrast, authors starting with a 1000 mg/d dose, increased to 2500 mg/d and, according to the gestational BMI, did not reduce the PE values (*p* = 0.58) (*p* > 0.01).^19^

NOTE: In this study found in the literature, there was no statistically significant value for body mass index reduction (*p* > 0.01) showed a decrease in the risk of PIH (RR = 0.37; 95% CI: 0.25 −0.57; *p* = 0.000) in pregnantwomen with PCOS.

### Outcomes of PE in Metformin versus Insulin Therapies in Pregnant Women with Gestational Diabetes Mellitus

Two recent meta-analyzes have confirmed that the effects of metformin on GDM have demonstrated consistent and/or favorable values both for the mother and for the newborn.^31^ ^32^ However, there is research that suggests more discussion about the subject. In the comparative studies using metformin versus insulin, the metformin was effective in reducing the weight of diabetic pregnant women, pointing to a higher BMI in pregnant women who administered insulin.^22^ ^33^

Study conducted in 2009 showed a strong relationship of increased BMI with chronic hypertension and PE during pregnancy.^34^ Likewise, the risks of PE in women with diabetes type I or II increased two to four times, respectively.^35^

Randomized clinical trials have confirmed the reduction of absolute and relative values for both PIH and PE incidence, when compared metformin with insulin. A research conducted in 2008 with mothers between 20 and 33 weeks of gestation, whose hospital criteriawere to initiate insulin, usedas a starting point a dose of 500 mg metformin once or twice daily, up to 2500 mg/d. Themaingoalwas to reachadequate glycemic levels. The PIH incidence values were 3.8% of the 363 mothers who received metformin compared with 6.21% of the 370 pregnant women who received insulin. Regading the incidence of PE, the values were 5.5% versus 7%, respectively.^22^

Another research developed, in 2012, with 500 mg/d and, from the third day after entering in the research, increased to 1000 mg/d. Metformin group (*n* = 110) percentages compared with control group (*n* = 107) were: 1.8% metformin versus 3.7% control for PIH, and 4.6% metformin versus 9.4% control for PE respectively.^23^ In the same year, other investigators in a similar procedure instituted an initial dose of 500 mg twice daily and, after two weeks, increased it to 1000 mg/d, increasing until reaching the appropriate target of the glycemic standards of the pregnant women, up to 2,500 mg/d, if necessary. Metformin group (*n* = 110) percentages compared with control group (*n* = 107) were: 1.8% metformin versus 3.7% control for PIH, and 4.6% metformin versus 9.4% control for PE.^24^

In the present meta-analysis, the values were significant in pregnant women with GDM only for PIH (RR = 0.53; 95% CI: 0.31–0.90; *p* = 0.018). Thus, although metformin has not demonstrated higher prophylactic values for PE in our literary findings, this drug has been confirmed to be safe during pregnancy and has improved the acceptance of metformin by pregnant women when compared to insulin.^22^

## Conclusion

It is important to clarify that the present study presented some limitations, such as a limited number of randomized clinical trials with the use of metformin in the PE outcomes. Another factor refers to the aspects related to the lack of information about the drug to be used both on the part of the examiner and of the person examined in the different researches. This would reduce the expectations of pregnant women and/or the placebo effect, which would allow a better understanding of the therapeutic effects of the drug related to its benefits and consequences. Metformin had better effects on milder hypertensive syndromes. The drug can gain space in preventive treatments for PE, once the dosages, GA and treatment time are better evaluated in contemporary studies. Compared with insulin, metmorfin has gained independence and suggests an adequate methodological strategy with an improved perspective of innovative and/or carefully progressive dosages during pregnancy, to avoid side effects and possible risks.
